# Automating Quality Assessment of Medical Evidence in Systematic Reviews: Model Development and Validation Study

**DOI:** 10.2196/35568

**Published:** 2023-03-13

**Authors:** Simon Šuster, Timothy Baldwin, Jey Han Lau, Antonio Jimeno Yepes, David Martinez Iraola, Yulia Otmakhova, Karin Verspoor

**Affiliations:** 1 School of Computing and Information Systems University of Melbourne Melbourne Australia; 2 Mohamed bin Zayed University of Artificial Intelligence Abu Dhabi United Arab Emirates; 3 School of Computing Technologies RMIT University Melbourne Australia; 4 Doctor Evidence LLC Santa Monica, CA United States

**Keywords:** critical appraisal, evidence synthesis, systematic reviews, bias detection, automated quality assessment

## Abstract

**Background:**

Assessment of the quality of medical evidence available on the web is a critical step in the preparation of systematic reviews. Existing tools that automate parts of this task validate the quality of individual studies but not of entire bodies of evidence and focus on a restricted set of quality criteria.

**Objective:**

We proposed a quality assessment task that provides an overall quality rating for each body of evidence (BoE), as well as finer-grained justification for different quality criteria according to the Grading of Recommendation, Assessment, Development, and Evaluation formalization framework. For this purpose, we constructed a new data set and developed a machine learning baseline system (EvidenceGRADEr).

**Methods:**

We algorithmically extracted quality-related data from all summaries of findings found in the Cochrane Database of Systematic Reviews. Each BoE was defined by a set of population, intervention, comparison, and outcome criteria and assigned a quality grade (high, moderate, low, or very low) together with quality criteria (justification) that influenced that decision. Different statistical data, metadata about the review, and parts of the review text were extracted as support for grading each BoE. After pruning the resulting data set with various quality checks, we used it to train several neural-model variants. The predictions were compared against the labels originally assigned by the authors of the systematic reviews.

**Results:**

Our quality assessment data set, Cochrane Database of Systematic Reviews Quality of Evidence, contains 13,440 instances, or BoEs labeled for quality, originating from 2252 systematic reviews published on the internet from 2002 to 2020. On the basis of a 10-fold cross-validation, the best neural binary classifiers for quality criteria detected risk of bias at 0.78 *F*_1_ (*P*=.68; R=0.92) and imprecision at 0.75 *F*_1_ (*P*=.66; R=0.86), while the performance on inconsistency, indirectness, and publication bias criteria was lower (*F*_1_ in the range of 0.3-0.4). The prediction of the overall quality grade into 1 of the 4 levels resulted in 0.5 *F*_1_. When casting the task as a binary problem by merging the Grading of Recommendation, Assessment, Development, and Evaluation classes (high+moderate vs low+very low-quality evidence), we attained 0.74 *F*_1_. We also found that the results varied depending on the supporting information that is provided as an input to the models.

**Conclusions:**

Different factors affect the quality of evidence in the context of systematic reviews of medical evidence. Some of these (risk of bias and imprecision) can be automated with reasonable accuracy. Other quality dimensions such as indirectness, inconsistency, and publication bias prove more challenging for machine learning, largely because they are much rarer. This technology could substantially reduce reviewer workload in the future and expedite quality assessment as part of evidence synthesis.

## Introduction

### Background

Systematic reviews, which aim to summarize the entirety of the available evidence on a specific clinical question, are the cornerstone of evidence-based decision-making in medicine. They are considered the strongest form of evidence because they analyze, aggregate, and critically appraise all relevant published evidence according to strictly defined protocols [1,2]. However, many factors impact our confidence in the overall effect estimate of that body of evidence (BoE), both at the level of individual primary studies (eg, limitations in design or conduct of a given study) and in aggregate across multiple studies (eg, the resulting sample size and number of events across studies, consistency of effects between studies, and amount of overlap between the study criteria and those specified in the clinical question). Given the role that systematic reviews play in shaping health policy guidelines and informing patient care, such limitations may ultimately be harmful and should, therefore, degrade our confidence in an intervention strategy. Thus, assessing the quality of evidence represents a critical step in the preparation of a systematic review and should be considered by downstream users of that evidence [3,4].

The construction of a systematic review is a complex and arduous process. Estimates of the time needed to complete a systematic review vary but can easily reach 1000 hours of (highly skilled) manual labor [5,6]. One component of this is the significant time needed to perform a quality assessment, with the assessment of risk of bias (RoB) alone (as one of several quality criteria) requiring >20 minutes per study [7]. Timeliness, cost of production, and availability of required expertise are the biggest obstacles to authoring systematic reviews to adequately support clinical practice and keeping them up to date [8-10].

Because of this inability to scale, further compounded by the ever-growing number of published medical studies [11], researchers have proposed to automate different steps of the reviewing process, including the automation of article classification, screening for primary studies, data extraction, and quality assessment [10,12]. In quality assessment, specifically, approaches that fully or partially automate the RoB estimation of individual studies included in a review have predominated [13-15]. These methods have used natural language processing (NLP) to extract the data elements from article text (typically the abstract) that are relevant to the review and that pertain to different bias criteria and then to classify the article as being either at low or high RoB. Such approaches have been shown to effectively speed up bias assessment in semiautomated settings, where humans are tasked with validating suggestions from machine learning (ML) models [16]. In this work, we address 2 limitations of the existing approaches and focus on the following:

1. Multiple aspects of the quality of medical evidence, instead of only the RoB.
2. Provision of a quality score for the entire BoE available for a specific clinical question, rather than rating the studies in isolation.

### Use Case

**Figure 1:**
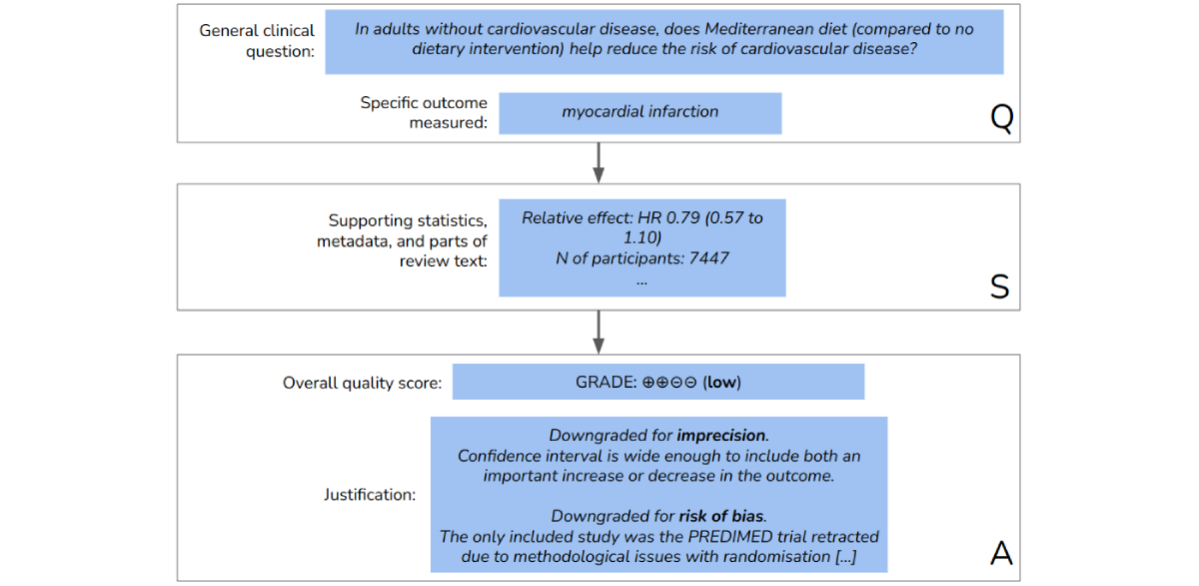
Assessment example, based on the systematic review Mediterranean-style diet for the primary and secondary prevention of cardiovascular disease [17]. Given a clinical question together with a specific outcome (Q), the task consists of making a quality assessment (A) by using the various data fields, as support (S), from the systematic review. GRADE: Grading of Recommendation, Assessment, Development, and Evaluation; HR: hazard ratio.

We set out to fill these gaps by introducing a data set for generalized quality assessment in systematic reviews and proposing ML methods to rate the quality and its associated components (criteria) for the entire BoE. We illustrate this task in Figure 1 [17]. From the automation perspective, different supporting data can be fed to the assessment tool, depending on their availability and the stage in the reviewing workflow at which the system is deployed. For example, a large part of the work done by the reviewers concerns meta-analysis, in which the collected data are summarized using statistical methods. At this stage, before the reviewers validate the quality of the evidence and prepare a narrative (which ultimately constitutes the main body of the review), an ML system can reach a (preliminary) quality judgment based on the clinical question and the available BoE. The predicted judgment and its justification can then support the reviewers in reaching a final decision. Our proposed model incorporates different sources of support data. In the empirical analysis, we inspect how the predictive performance is affected by removing a class of features (or a source of data), such as all textual features that represent the summarized narrative. With this, our goal is to better understand how the system would perform at a specific stage in the reviewing process when restricted support data are available to the system. We further reflect on this in *Implications for the preparation of systematic reviews* section.

As argued for in the context of RoB analysis [13], an accurate system for automated grading could expedite and enhance the critical appraisal of medical evidence, freeing up researcher time to concentrate on thoughtful evidence synthesis, and ultimately would help keep systematic reviews up to date. *Our work expands the scope of quality-of-evidence assessment automation to consider a fuller range of quality criteria and their synthesis across multiple studies.* We implemented EvidenceGRADEr available in the study by Soboczenski et al [16].

## Methods

### Background

#### Cochrane Reviews

Cochrane reviews are high-quality, independent systematic reviews of research in health care and health policy that are published in the Cochrane Database of Systematic Reviews (CDSR) [18]. They focus on synthesizing the evidence as found in randomized controlled trials. The format of Cochrane reviews is to a large extent standardized [19] and is described in detail in the reporting guidelines [2].

#### Grading of Recommendation, Assessment, Development, and Evaluation Quality Assessment Framework and Its Place in Cochrane Reviews

Various frameworks have been used to appraise the quality of a BoE in systematic reviews [20]. Perhaps, the most popular scheme that supports systematic appraisal is Grading of Recommendation, Assessment, Development, and Evaluation (GRADE) [3], which has been adopted by CDSR. A major advantage of GRADE is that it leads to more transparent judgments regarding the quality of evidence [21,22]. The quality of evidence for individual outcomes according to GRADE is scored based on the following five key quality criteria:

1. RoB (pertaining to study limitations).
2. imprecision of the estimated effect (risk of random errors, especially in the presence of a small number of studies, with a small effect size and large CIs).
3. inconsistency of evidence (unexplained dissimilarity of point estimates between studies).
4. indirectness of evidence (uncertainty about the applicability of the evidence to the relevant clinical question).
5. likelihood of publication bias (likelihood of missing evidence, especially with many small, industry-funded studies).

In GRADE, each BoE obtained from randomized control trials starts with the highest certainty (high) in the quality of evidence and remains as such, assuming there are no concerns in any of the GRADE factors listed above. In contrast, the certainty can be downgraded for any of the quality factors, and the overall GRADE score can be adjusted correspondingly (from high to moderate, low, and very low). Usually, the certainty rating falls by 1 level for each factor, up to a maximum of 3 levels. If there are very severe problems for any 1 factor (eg, when assessing RoB, all studies were unconcealed, unblinded, and lost over 50% of their patients to follow-up), evidence may fall by 2 levels because of that factor alone.

In Cochrane reviews, the information about the quality of evidence is presented in summary of findings (SoF) tables, which summarize the main findings. Justifications for any GRADE adjustments are typically provided in the SoF table in the form of a footnote to the relevant entries. Apart from the information related to the quality of evidence, the summaries include the clinical question given as population, intervention, comparison, and outcome (PICO) descriptors; quantitative data pertaining to the studies constituting the BoE; and the magnitude of effect of the interventions examined. The GRADE assessment reported in SoF tables is outcome centric, that is, rating is performed for each outcome deemed important for clinical decision-making, and quality may differ from one outcome to another. Most reviews contain a single SoF table, but in cases where there is more than one major dimension of comparison, or substantially different populations, there may be multiple such tables. In addition to SoF tables, quality-related information can be summarized in other parts of a systematic review, normally as a narrative. According to Thornton et al [22], assessing the quality of evidence for one outcome takes a median of 30 minutes for professional reviewers with several years of experience. The GRADE assessment scheme subsumes the standard RoB tool developed by Cochrane [7,23].

#### Consistency of GRADE Ratings

Because we approach the task of automated quality assessment by constructing a data set from quality judgments and justifications made by humans, it is important to understand the reliability of those annotations. Several studies have investigated the reliability of annotations using GRADE [20,21,24-28]. Overall, the reliability was found to be variable, depending on factors such as the number of raters, their experience, and the quality criterion evaluated (when the raters were asked to assign not only the overall grade but also the downgrading reason). We summarize the findings of these studies in Multimedia Appendix 1 [20,21,24-28].

Briefly, the interrater reliability for assigning an overall quality score according to GRADE in Cochrane reviews is likely to range between fair and substantial, depending on criteria such as the experience level of annotators. Few studies have addressed the agreement for the individual quality criteria. On the basis of the study by Thornton et al [22], imprecision and RoB appear to be the 2 quality criteria that are most consistently assigned, whereas indirectness requires the most judgment and is hence elusive. High consistency among RoB annotations have also been reported by Berkman et al [25]. Multimedia Appendix 2 and Multimedia Appendix 3 provide the analysis of RoB consistency in our data [13].

#### Modeling: Prior Work

Efforts to automate the quality assessment of medical evidence have largely been geared toward predicting the RoB, and there has been little work on other quality components. We summarize the existing work in the following sections.

A series of studies [13,14,29,30] have proposed an approach to automating RoB assessment in systematic reviews, in which an ML system determines whether the study results may be affected by biases (eg, poor randomization or blinding) and provides supporting sentences from the study abstract. The authors adopted the standard Cochrane RoB Tool that formalizes 7 common types of bias. Their approach of obtaining data labels is similar to ours; they refer to it as distant supervision, that is, using data from CDSR to pseudoannotate a corpus of 2200 clinical trial reports. The RobotReviewer model developed on this data achieves an accuracy of approximately 70% when categorizing articles as at low or high and unclear RoB.

A corpus of approximately 1100 abstracts with metadata [31,32] has been used to predict 3-tier quality grades and specify the strength of recommendation of a BoE as strong, moderate, or weak. The accuracy with fully automatic feature extraction ranged between 51% and 60%, depending on the machine learner, with a maximum of 64% when using a combination of different models. However, on this data set, the human annotators only achieved a Cohen κ agreement of approximately 0.5. Only publication metadata features (eg, type, year, venue, and title) and word n-grams from abstracts were used in the modeling. The authors adopted the Strength of Recommendation Taxonomy framework for grading the strength of recommendation [33], which does not offer rationales for the overall quality decisions, unlike GRADE. The work frame determines the strength of evidence as a simple sum of individual scores assigned to primary studies based on attributes such as journal type, publication type, and publication year. Our work extends this by (1) performing an assessment using synthesized data from systematic reviews and not only the primary studies, (2) using features beyond publication metadata, and (3) predicting the component quality criteria that give rise to the overall rating.

Semiautomated Quality Assessment Tool [34] provides an assessment of the overall quality of evidence by formalizing the structure provided by the GRADE framework in a logic model and by assigning a specific weight to each of the different items considered by GRADE. This approach does not rely on extracting source data from the reviews and using ML to predict the quality of evidence but still expects that the checklist questions that inform the GRADE quality criteria are answered by humans.

Another related line of research [35-38] seeks to identify articles containing high-quality clinical evidence based on the annotations of scientific rigor from the study by Byczyńska et al [38]. They created a collection of approximately 49,000 MEDLINE documents of which approximately 3000 were identified as methodologically rigorous and the rest as nonrigorous. It is unclear from the description of the data set if the authors received any other guidance apart from the brief description of the factors [39] that led to a positive evaluation of methodological rigor. In contrast, GRADE offers substantial guidance to authors performing quality assessments, as well as continuous updates of their guidelines. The task of scientific rigor classification assigns a single binary label to an article and hence does not consider individual outcomes or the entire BoE for a clinical question, as we do in this paper.

### Data Set Construction and Quality Control

#### Overview

We built our data set from the snapshot of the CDSR on June 26, 2020, which contained 8034 reviews. We translated each review into a JSON representation, with structured representations of the review metadata, textual parts of the review (abstracts and summaries), SoFs, and characteristics of the primary studies. We decided to use JSON for its human readability and the concise format, which has little boilerplate content and therefore results in smaller file sizes compared with other formats, such as XML.

**Figure 2:**
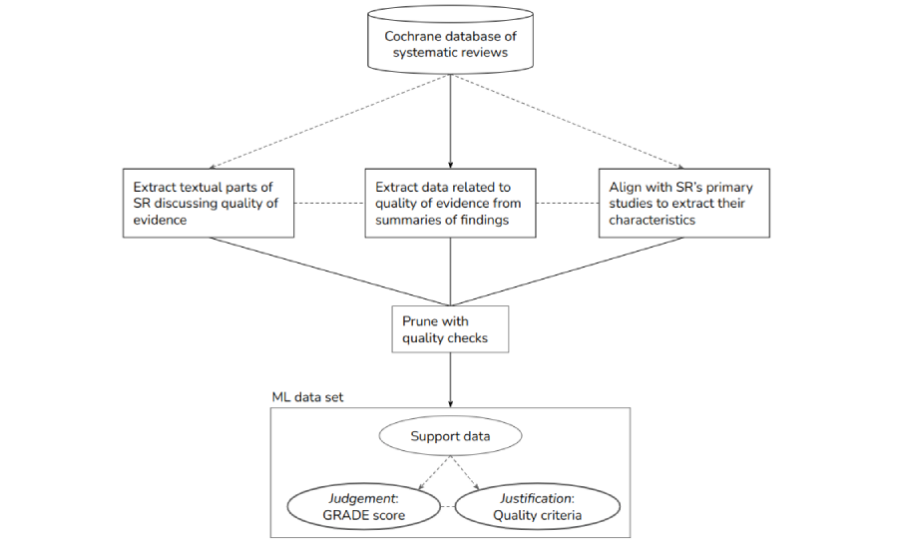
Schematic of our data construction approach. GRADE: Grading of Recommendation, Assessment, Development, and Evaluation; ML: machine learning; SR: systematic review.

Figure 2 provides an overview of the data construction process. From this initial structured representation of Cochrane reviews, we created our final data set in which all data fields corresponding to individual features were included in a CSV file. The complete list of data fields (that we also used in our experiments) is shown in Table 1, and descriptive statistics of the textual files are given in Multimedia Appendix 4. Although the extraction of GRADE scores from SoFs is trivial, justifications (reasons for downgrading) are not provided consistently. We therefore had to search for these reasons in the footnotes of each SoF. As the authors used different terms to refer to a particular downgrading category, we manually constructed a simple mapping between a term and the triggered downgrading category (eg, heterogeneity→inconsistency). Once the criteria were extracted, we implemented a straightforward filter to increase the accuracy of the quality-related labels in our data set as follows. We kept track of the total number of downgrading steps over all downgrading categories applicable for each data instance and compared this count to the overall GRADE quality score (0=high to 3=very low). Whenever the 2 did not match, we removed that data instance. For example, if the authors discount for imprecision by 2 steps and for RoB by 1, the expected GRADE score is very low because the level of quality was downrated 3 times. Instances where misalignment was observed were excluded. Finally, we skipped this check for all high-quality evidence because, by default no reason exists (or should exist) that could undermine the quality of evidence. We refer to the resulting data as the CDSR Quality of Evidence (CDSR-QoE).

**Table 1:** Data fields in Cochrane Database of Systematic Reviews Quality of Evidence data set.

Data field	Type	Data source
review type	cat	Metadata
medical area (topics)	cat	Metadata
type of effect	cat	SoF^a^
Year	num	Metadata
# of SoFs	num	SoF
# of participants	num	SoF
upper CI	num	SoF
lower CI	num	SoF
# of outcomes	num	SoF
relative effect	num	SoF
# of studies	num	SoF
# of included studies	num	Metadata
# of ongoing studies	num	Metadata
# of other studies	num	Metadata
# of additional studies	num	Metadata
# of excluded studies	num	Metadata
outcome	text	SoF
abstract conclusion	text	Body text
plain language summary	text	Body text
full abstract	text	Body text
authors’ conclusions	text	Body text
methods^b^	cat	Primary studies
judgement and justification for each RoB^c^ component^b^	cat	Primary studies
# of “low,” “high,” or “unclear” for each RoB component^b^	num	Primary studies
proportion of “high” for each RoB component^b^	num	Primary studies

^a^SoF: summary of findings.

^b^Items represent additional support features derived from primary studies.

^c^RoB: risk of bias.

#### Supplementary Partially Labeled Data for Quality Criteria

Although we filtered out instances in which there was a misalignment between the overall GRADE and the total number of downgrading steps, manual examination of the data suggested that the issue occurred predominantly because of a failure on the part of our extraction scripts to detect one or more reasons (ie, because of the imperfect recall of our extraction methodology). Nevertheless, in such cases, we have a subset of the downgrading reasons that were detected. These instances can be used as partially labeled data to model quality criteria. That is, in addition to the fully labeled (in terms of overall BoE and quality criteria) CDSR-QoE data set, D = {(x_i_, y_i_)}_i=1_^N^ ; y ∈ {0, 1}—where x indicates the vector of input features, y the presence of a downgrading reason, and N the original number of instances; we also include positive instances that are partially labeled for quality criteria downgrades, D^S^ = D ∪ D’ — where D’ = {(x’_i_ , y’_i_ )}_i∈I’_, I’ := {i ∈ 1...K : y’_i_ = 1}—from K instances filtered out during the construction of the CDSR-QoE data set. In doing so, we reduced the positive-negative label imbalance for the less frequent classes (inconsistency, indirectness, and publication bias). The resulting changes in the label distributions are shown in Table 2. We only supplemented the training data during each fold of the cross-validation trial, leaving the development and test sets intact. We refer to this data set as CDSR-QoE-supp.

**Table 2:** The effect of data supplementation on the distribution of quality criteria. The number of positive instances introduced with supplementation is contrasted to the number of positive and negative instances in Cochrane Database of Systematic Reviews Quality of Evidence (CDSR-QoE).

	Positive (CDSR-QoE)	Negative (CDSR-QoE)	Positive (CDSR-QoE-supp)
RoB^a^	6433	4336	9013
Imprecision	6028	4741	11,273
Inconsistency	1759	9010	2664
Indirectness	1170	9599	2073
Publication bias	519	10,250	881

^a^RoB: risk of bias.

#### Alignment to Primary Studies

**Figure 3:**
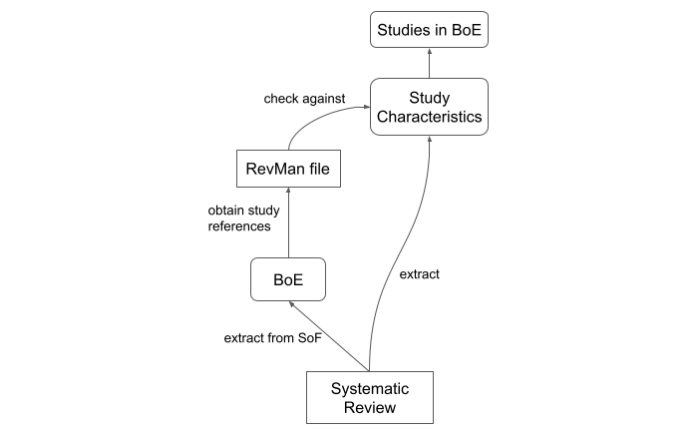
Diagram illustrating the procedure for obtaining studies relevant to a single body of evidence. These studies typically represent a subset of all studies included in the review. BoE: body of evidence. SoF: summary of findings.

To explore the expected contribution of including low-level study characteristics when assessing entire BoEs for quality, we aligned the data set to the underlying primary studies. The Cochrane reviews contain a list of all studies (together with their characteristics) that form the basis for evidence synthesis in that systematic review. However, the quality-evaluated outcomes in SoFs lack alignment with the subset of included studies that form the evidence base. Therefore, we retrieved the relevant studies for each clinical question from the review preparation software files (Rev Man; the Cochrane Collaboration), which contained the original data pertaining to the performed meta-analysis, including the relevant study references. We then attempted to match these with those included in the *Characteristics* section of the review. In cases where we failed to find an exact match in the *Characteristics* section, we performed minimum edit distance matching at the character level while ensuring that certain other conditions matched exactly (type and size of the effect and number of studies). Using both exact and minimum edit distance matching, we were able to retain 27% of the original data instances. We provide a graphical representation of the alignment procedure in Figure 3.

Broadly speaking, the alignment with the primary studies provides 2 types of study characteristics: the method description and the judgments and justification for the study-level RoB (Table 1). We applied the alignment procedure to the CDSR-QoE-supp data set and referred to it as CDSR-QoE-aligned.

### Validation of Data Set Construction

To perform an external validation of our extraction procedures used in data set construction, we used human annotations from the small-scale study by Wilczynski et al [39], in which various elements were manually extracted from SoFs. Although a larger study was carried out by Conway et al [40], we were unable to obtain their data. The data set in the study by Wilczynski et al [39] contains 103 instances (reviews) on anesthesia, critical care, and emergency medicine from the CDSR. For these, the GRADE score existed for the first primary outcome reported in the summary. We evaluated our extracted GRADE score, reasons for downgrading, and the number of studies and participants by performing exact matching against their data set.

For extracting the number of studies, we initially observed an accuracy of 0.69. Upon manually checking the cases believed to be incorrect, we found that most of them were not errors but resulted from different review versions or the inclusion of an outcome that was not the first in the table. After correcting for these issues, the estimated accuracy reached 0.94. We carried out the same analysis for the number of participants, GRADE score, and downgrading reasons, obtaining similar results (for GRADE, 0.93 accuracy; for downgrading reasons, 0.96). Importantly, the few mistakes that we encountered were the results of failing to successfully extract a value (leaving an empty field), so we excluded these instances from the final data set.

### Summary Statistics for the CDSR-QoE Data Set

**Figure 4:**
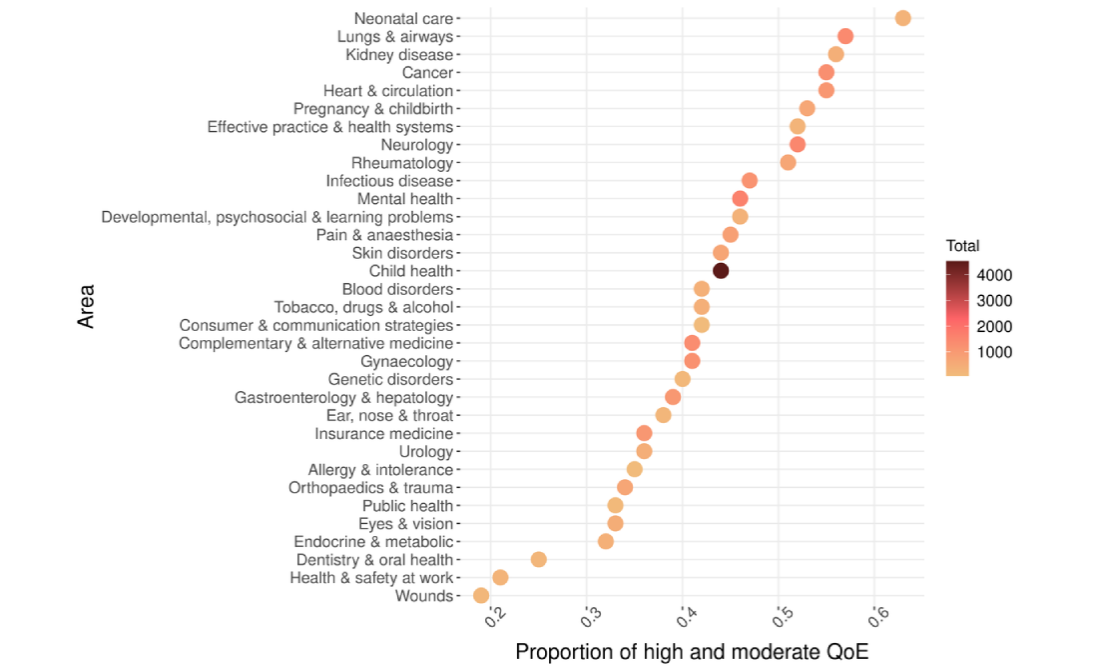
Distribution of quality of evidence (high- and moderate-quality vs all) across systematic-review areas. The number of data instances characterized by each medical area varies (min=65, max=4526, mean=768), and is illustrated by color-coding the points. QoE: quality of evidence.

Some statistics from the final CDSR-QoE data set are shown in Table 3. One interesting observation is that among >13 k instances (BoEs) making up the data set, most of the evidence (54%) is of (very) low quality, and the quality is high (ie, includes no apparent reason for downgrading) only 14% of the time. For a single data instance, >1 downgrading reason can be assigned (affecting the overall GRADE score proportionately, as discussed in aforementioned sections). The 2 most frequently co-occurring reasons were the RoB and imprecision, which occurred together in 30% of all instances. We visualized the relationship between the review area and the proportion of evidence of higher quality (high or moderate) in Figure 4. The percentage of higher-quality evidence is <50% for most medical areas, and for some areas (eg, dentistry), it is <25% of all evidence. This means that clinicians very often do not have firm evidence to support the effectiveness of a large number of interventions across these medical areas.

**Table 3:** Summary statistics for the Cochrane Database of Systematic Reviews Quality of Evidence data set.

Data set		Values
**BoE^a^ (N=13,440), n (%)**		
	High quality	1909 (14.2)
	Moderate quality	4232 (31.5)
	Low quality	4562 (33.9)
	Very-low quality	2737 (20.4)
**Factors affecting quality, number of annotations, n**		
	Risk of bias	7969
	Imprecision	7377
	Inconsistency	2208
	Indirectness	1388
	Publication bias	667
Number of reviews, n		2252
Number of studies per review, mean (SD)		20 (25)
Number of studies, n		40,375
Number of summaries of findings per review, mean (SD)		2 (1.8)

^a^BoE: body of evidence.

### ML Approach

**Figure 5:**
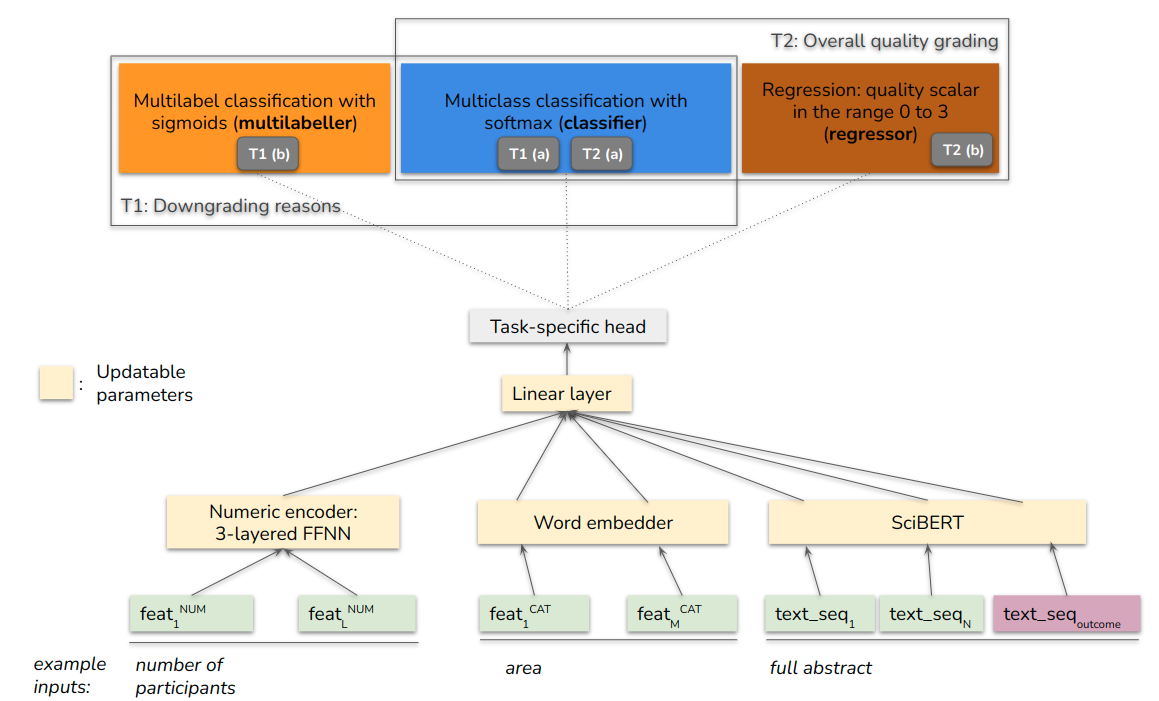
Our feature-rich modeling approach to automated quality assessment. The actual form of the output layer depends on the task. The schematic shows all possible task-specific heads, but we train and evaluate the model for each task (T1 [b], T1 [a], T2 [a], and T2 [b]) independently. FFNN: feed-forward neural network. NUM: numerical. CAT: categorical.

In this section, we introduce our approach for predicting the quality of evidence. Our goal was to obtain a solid baseline system for our task that has the flexibility to accommodate heterogeneous inputs (numerical, categorical, and textual) and can be used for different modeling subtasks (as explained at the end of this section). Therefore, we implemented a neural model that uses different encoders to represent the heterogeneous input features, then aggregated these representations with a linear layer, and finally predicted a label (Figure 5). Apart from the output layer, we maintained the same model architecture for both tasks, that is, predicting the downgrading reasons and assigning an overall quality grade. All numeric inputs were scaled using min-max normalization, and all encoder outputs before linear aggregation were layer normalized (Ba, JL, unpublished data, July 2016). The trainable parameters of the model consist of the linear aggregation layer and 3 feature encoders. The encoder for numeric inputs is a 3-layered feedforward neural network; the categorical inputs were embedded using randomly initialized vectors; and the unstructured textual inputs were represented using SciBERT, a pretrained transformer-based language model [41,42]. Although categorical inputs could be encoded in principle with a pretrained language model, we considered such an approach excessive. There is little need for context sensitivity within categorical inputs, and the approach would lead to unnecessary computational costs. By contrast, a simpler solution using sparse or one-hot encoding might fall short of relating different but related feature categories (eg, medical areas). For textual inputs, our decision to use SciBERT over BERT was based on preliminary experimentation, which showed better performance of SciBERT on our development sets. This can be explained by the fact that the model is trained largely on biomedical texts, whereas BERT uses a general-domain corpus. More information on the impact of specific language model instantiations can be found at the end of the section titled *Predicting individual quality criteria*. Our encoding approach using SciBERT works as follows: it first tokenizes text into word pieces using a model from HuggingFace's transformers library, encodes each text sequence separately, updates SciBERT parameters, and finally takes the hidden layer outputs at the sequence-level classification level as the representations, which are then concatenated as the output representation. We use the outcome specified in the SoF to ground the BoE that is being assessed (quality is likely to differ with respect to the outcome studied within the same review).

The form of the output layer depends on the task, which is detailed in Textbox 1.

In our comparison of results, we included various baselines. The two trivial approaches include (1) a predictor that selects a GRADE score or quality criterion at random using a discrete uniform distribution, and (2) a majority-class baseline that uniformly predicts the most frequent class. That is, for the GRADE score, the model always predicts low, and for quality criteria, the model outputs RoB+Imprecision (the most frequent label combination in the training set).

In addition, we trained a logistic regression (LR) model with 3 different input representations: (1) numerical features; (2) numerical and categorical features using bag-of-word counts; and (3) numerical, categorical, and textual inputs represented using bag-of-word counts. Note that at a high level, (3) resembles EvidenceGRADEr but represents the different input features in a simpler way. We trained and evaluated the LR models on the 2-tier (binary) quality-grading task.

We report the exact experimental settings for our models in Multimedia Appendix 5 (Kingma DP, unpublished data, December 2022).

**Overview of tasks.**

- T1 downgrading reasons
  (a) Modeled as a series of independent binary classification problems, one for each quality criterion.
  (b) Modeled jointly as one 1 multilabel classifier for all quality criteria, in which each linear layer output that corresponds to a downgrading reason is passed through the sigmoid function, and then the prediction corresponds to those units that fired. To counteract the class imbalance in this case, we weight the examples inversely proportionally to their class frequency when calculating the binary cross-entropy loss.
- T2 overall quality grading
  (a) Modeled as multiclass classification using cross-entropy loss during training.
  (b) Modeled as regression, in which the model assigns a scalar corresponding to the quality grade, with 0 corresponding to very low, 1 to low, 2 to moderate, and 3 to high quality. In this case, we use the mean-squared error loss.

### Evaluation Details

We trained and evaluated our models using 10-fold cross-validation, keeping one-tenth of the data for validation, one-tenth for testing, and the rest for training. When splitting into different folds, we ensured that all BoEs pertaining to the same systematic review were kept within the same fold to prevent any leakage of similar instances between training and testing partitions [43].

During the evaluation, we reported per-class *F*_1_*-*scores, as well as the macroaverage over those scores by simply averaging them with equal weight. The reported precision and recall values for all classes were calculated analogously. All scores represent the averages over the 10 trials of cross-validation.

We used a single set of hyperparameters across all cross-validation iterations. Although the textual encoder used the default BERT settings, the remaining hyperparameters of the model architecture (Multimedia Appendix 5) (Kingma DP, unpublished data, December 2022) were tuned on the development set of the first cross-validation fold.

## Results

### Predicting Individual Quality Criteria (T1)

The overall quality of evidence according to GRADE represents a single, discrete categorization of the quality per outcome, informed by the underlying quality criteria. Although the GRADE score should follow these criteria in a straightforward manner, it represents an additional abstraction and potentially another source of arbitrariness. Therefore, we focus our discussion first on the results obtained for individual quality criteria. Table 4 summarizes the results for binary classifiers (T1 [a]), trained and evaluated separately for each quality criterion, and for a multilabelling approach (T1 [b]). With the full model using all the input features listed in Table 1 on CDSR-QoE, the performance is >0.7 *F*_1_ for 2 criteria: RoB and imprecision. We note that our RoB results are comparable to the accuracy observed for RobotReviewer (around 70%) [14].

The remaining GRADE criteria proved more elusive to the model, with especially low recall. Note that these classes represent the least frequently applied downgrading reasons in our data set; hence, the relative sparsity of the positive instances is the greatest. We would expect the performance to improve in situations where more positive instances are available. Indeed, we see that when supplementing our data set with partially labeled data (CDSR-QoE-supp), the performance improves across the board, with increased precision and recall. The results described so far concern the full use of the support data. If we remove all textual inputs (“−txt”), and preserve the numerical and categorical features, the performance drops for all criteria, meaning that the different textual summaries included in our predictor play an important role, most notably for indirectness and publication bias. This means that the reviewers encode information in the narrative that is otherwise not present in a structured form and that this complementary signal in the textual inputs can be picked up effectively by the classifier to inform its decision. That said, the model that is deprived of any textual inputs performs reasonably well and could be actionable in practice, especially for the most common quality criteria, that is, the RoB and imprecision.

As a side effect, we explored the importance of choosing a particular instantiation of a pretrained language model as our text encoder. Therefore, we replaced the pretrained SciBERT language model with two alternatives: (1) a general BERT model (BERT-base-uncased) [42] and (2) a BioMed-RoBERTa-base model [44], which is based on the RoBERTa-base architecture (Liu, Y, unpublished data, July 2019) and pretrained on full scientific papers from the Semantic Scholar corpus [45]. We found that using the general BERT model resulted in slightly decreased *F*_1-_scores for the quality criteria, whereas BioMed-RoBERTa-base improved over SciBERT for RoB, indirectness, and inconsistency, but not for imprecision and publication bias. The overall differences in the scores were small, as shown in Multimedia Appendix 6 [20,21,24-28].

We observed some variation in results between different cross-validation trials (shown in the subscript in Table 4), which tended to be smaller for the classes with more data (RoB and imprecision) and larger for the 3 less frequent classes.

As a single data instance can be characterized according to multiple downgrading criteria, we also evaluated the multilabelling performance on the predictions of all binary models. We find that the exact match accuracy in this case is 0.29 (for the model using supplementary data from CDSR-QoE-supp), counting as correct only those instances in which all reasons were correctly predicted. This corresponds to a microaveraged *F*_1_-score of 0.68 (0.53 when macroaveraging). These results are superior when compared with the multilabelling approach (multilabeller in Table 3), which, despite the ability to capture interactions between the labels, achieves an overall accuracy of 0.27 (0.56 micro- *F*_1_; 0.48 macro- *F*_1_).

**Table 4:** Test set results for classification of reasons for downgrading the quality of evidence (T1), reporting macroaveraged F1 together with median absolute deviation (subscripted) across all trials of 10-fold cross-validation.

Data setup and model			GRADE^a^ downgrading criteria								
			RoB^b^, *F*_1_		Imprecision, *F*_1_		Inconsistency, *F*_1_		Indirectness, *F*_1_		Publication bias, *F*_1_
**CDSR-QoE^c^**											
	Random	0.50_.02_		0.48_.02_		0.23_.02_		0.17_.02_		0.08_.02_	
	Majority	0.74_.02_		0.71_.02_		0.00_.00_		0.00_.00_		0.00_.00_	
	Classifier^d^	0.75_.02_		0.72_.02_		0.09_.02_		0.11_.02_		0.02_.00_	
	Multilabeller^e^	0.74_.02_		0.64_.02_		0.25_.02_		0.25_.13_		0.14_.13_	
**CDSR-QoE-supp**											
	Classifier	0.78_.02_		0.75_.02_		0.31_.02_		0.41_.02_		0.39_.12_	
	Multilabeller	0.64_.02_		0.59_.02_		0.29_.02_		0.47_.02_		0.44_.21_	
	Classifier, −txt^f^	0.72_.02_		0.74_.02_		0.26_.02_		0.24_.02_		0.19_.13_	
**CDSR-QoE-aligned^g^**											
	Classifier	0.71_.02_		0.69_.02_		0.47_.02_		0.28_.26_		0.05_.00_	
	Classifier, +PS^h^	0.74_.02_		0.66_.02_		0.51_.02_		0.30_.10_		0.13_.00_	

^a^GRADE: Grading of Recommendation, Assessment, Development, and Evaluation.

^b^RoB: risk of bias.

^c^CDSR-QoE: Cochrane Database of Systematic Reviews Quality of Evidence.

^d^For classifier (T1 [a]), each reason type is independently trained and evaluated in a binary setting (eg, “risk of bias” vs “other”).

^e^For multilabeller, a single model is tasked with predicting multiple reasons (T1 [b]).

^f^−txt: with removed textual inputs.

^g^As CDSR-QoE-aligned is smaller and with different test sets compared with CDSR-QoE, the results are not directly comparable between the 2.

^h^+PS: with added primary study–related features.

### Can Low-Level Signal From Primary Studies Further Improve Automated Quality Assessment?

CDSR-QoE-aligned provides us with links to the characteristics of primary studies that form the BoE. The effect of adding primary study–related features (+PS) was positive for RoB (Table 4). This was expected because the bulk of the added features (bottom of Table 4) relate to RoB components. In fact, one might expect the advantage of adding RoB judgments and justifications to be even more pronounced, as these low-level RoB decisions have already been made by the reviewers. However, the judgments that are input to our model still only belong to individual RoB components and not to an overall RoB judgment. In addition, they are concerned with only 1 primary study, whereas the model needs to assign a RoB decision for the entire BoE. These 2 factors may explain why the classifier failed to attain a larger improvement in the presence of low-level RoB judgments. The effect was also positive for publication bias, which possibly correlates with specific components of RoB or could be informed by the study method description. We observed increased *F*_1_-scores for inconsistency and indirectness; however, the variability between the cross-validation trials was higher (largely because of data sparsity).

Because of the difficulty in obtaining alignments to primary studies, we had to perform our analysis on a smaller subset of the original data, as explained in the *Data Set construction and quality control* section. Although we found the incorporation of primary studies to be beneficial, a larger-scale evaluation and exploration of the impact of other features from the primary studies (eg, textual abstracts and metadata such as journal titles of the published articles) are warranted in the future to offer a more comprehensive view.

### Predicting Overall Quality (T2)

We now shift our discussion to the overall quality grading, which we analyze at 2 granularity levels. The first maintains the original 4-tier GRADE scoring scheme, whereas the second merges the levels to obtain a binary score. In the 2-tier case, we merge high and moderate and low and very low. This is motivated by how quality assessment informs the guideline development. Guideline authors will form the recommendations based on their confidence in all effect estimates for each outcome that is considered critical to their recommendation and the quality of evidence. Typically, a strong recommendation is associated with high, or at least moderate, confidence in the effect estimates for critical outcomes. Conversely, GRADE discourages guideline panels from making strong recommendations when their confidence in estimates of effect for critical outcomes is low or very low [46]. The results in Table 5 show that our model improves significantly over trivial baselines. The classification approach yielded better results on *F*_1_ metrics and achieved stable performance over the 4 levels of GRADE (∼0.5 *F*_1_). In comparison, the regression approach made more mistakes for the outermost classes but achieved, on average, a smaller absolute error (0.62). In the simplified binary task, our classifier achieved an *F*_1_-score of 0.74. From the LR baseline results we see that it is important to include all 3 input categories (numerical, categorical, and textual) and that a more complex representation approach of EvidenceGRADEr of these categories is warranted (0.74 vs 0.66 *F*_1_). Nevertheless, the LR approach clearly outperformed the 2 trivial baselines, regardless of the included input categories.

**Figure 6:**
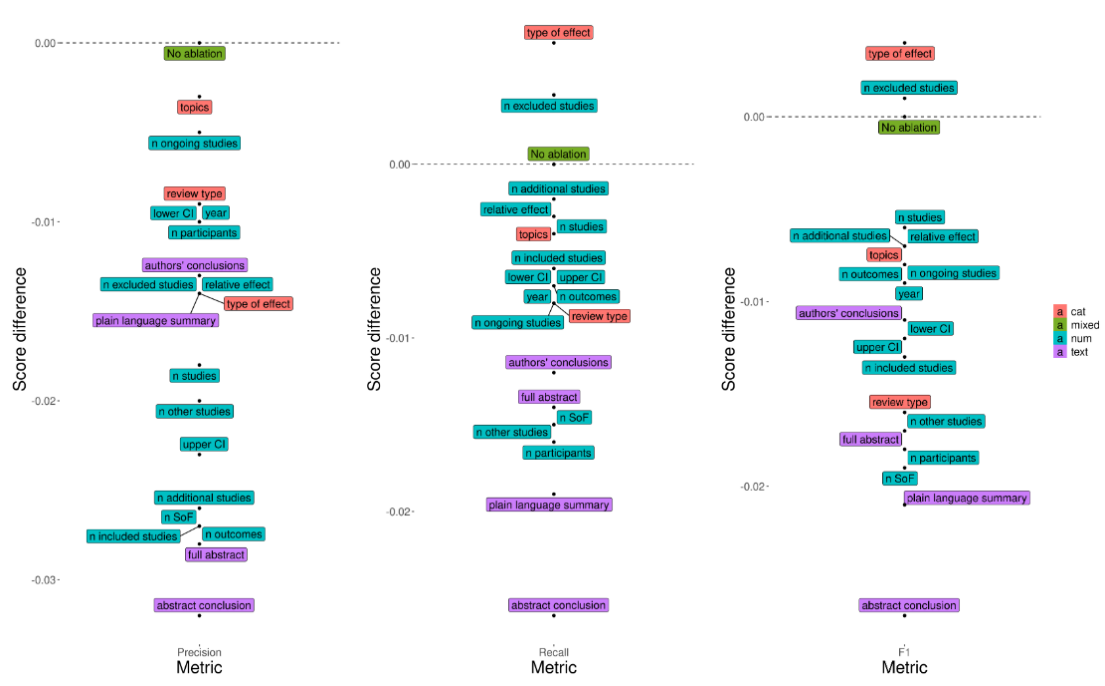
Feature ablations over the different metrics for predicting the overall Grading of Recommendation, Assessment, Development, and Evaluation (GRADE) score. A feature is considered important if its removal results in a large drop in performance (is located lower in the plot). The scores on y-axes represent the difference to the model with no ablation (the dashed horizontal line). SoF: summary of findings. NUM: numerical. CAT: categorical. CI: confidence interval.

To better understand which systematic review inputs play an important role in the overall quality assessment, we performed an ablation analysis using the 4-tier GRADE classifier. We removed 1 feature at a time and noted the changes in the scores for each of precision, recall, and *F*_1_. As shown in Figure 6, all feature types (numerical, categorical, and textual) contribute to the quality of prediction. The removal of certain textual features has the greatest impact on performance (especially abstracts and conclusions). We also found that the type of effect and the number of excluded studies harm the performance (in the case of recall and *F*_1_) and could be removed from the model. On the basis of the *F*_1_ plot, there appeared to be no clearly redundant features, the removal of which would leave the score unchanged.

Finally, considering that medical evidence (also, the prevalence of higher-quality evidence) is nonuniformly distributed across medical areas, as shown in Figure 4, the predictive performance on different medical areas may also be variable. This is an important question from the perspective of practical application, which we intend to explore separately in the future, owing to its complexity. To provide an impression of the out-of-domain generalizability of our system, we conducted an experiment using mental health as the selected area. Although the quality of 4-tier predictions in mental health alone equals the average performance over all areas (0.49 *F*_1_), the situation becomes interesting when we look at how well the model generalizes when it is trained on all areas *except* mental health and is then evaluated on mental health *only*. This results in a decreased *F*_1_ (from 0.49 to 0.43); however, in binary grading, the performance remains the same, meaning that only finer-grained distinctions are negatively affected. Additional analyses related to the generalizability can be obtained from the authors.

**Table 5:** Results on the test set for overall quality scoring using Grading of Recommendation, Assessment, Development, and Evaluation (T2) on Cochrane Database of Systematic Reviews Quality of Evidence, averaged over 10 folds, with median absolute deviation in subscript.

Setup and model		MAE^a^	*P* value	R	*F*_1_	Scores					
						*F*_1_ high	*F*_1_ moderate	*F*_1_ low	*F*_1_ very low	*F*_1_ positive	*F*_1_ negative
**4-tier**											
	Random	1.20_.02_	.25_.02_	0.25_.02_	0.25_.02_	0.23_.01_	0.30_.03_	0.28_.04_	0.18_.06_	N/A^b^	N/A
	Majority	0.80_.06_	.08_.01_	0.25_.00_	0.13_.01_	0.00_.00_	0.00_.00_	0.51_.04_	0.00_.00_	N/A	N/A
	LR^c^-n^d^	1.02_.02_	.26_.01_	0.27_.01_	0.25_.01_	0.08_.03_	0.39_.05_	0.41_.04_	0.12_.03_	N/A	N/A
	LR-nc^e^	0.96_.05_	.37_.01_	0.31_.01_	0.29_.02_	0.09_.04_	0.42_.04_	0.44_.02_	0.21_.05_	N/A	N/A
	LR-nct^f^	0.89_.05_	.46_.01_	0.37_.02_	0.36_.02_	0.28_.08_	0.47_.03_	0.38_.05_	0.31_.06_	N/A	N/A
	Classifier	0.77_.05_	.54_.04_	0.49_.03_	0.49_.03_	0.47_.06_	0.53_.08_	0.51_.05_	0.46_.06_	N/A	N/A
	Regressor	0.62_.02_	.56_.07_	0.42_.03_	0.42_.04_	0.30_.13_	0.50_.04_	0.52_.03_	0.37_.08_	N/A	N/A
**2-tier** ^g^											
	Random	N/A	.53_.00_	0.53_.00_	0.52_.00_	N/A	N/A	N/A	N/A	0.52_.03_	0.47_.02_
	Majority	N/A	.27_.02_	0.50_.00_	0.35_.02_	N/A	N/A	N/A	N/A	0.00_.00_	0.70_.03_
	LR-n	N/A	.58_.02_	0.57_.02_	0.57_.02_	N/A	N/A	N/A	N/A	0.50_.05_	0.64_.03_
	LR-nc	N/A	.64_.02_	0.63_.03_	0.63_.02_	N/A	N/A	N/A	N/A	0.57_.05_	0.69_.03_
	LR-nct	N/A	.68_.02_	0.67_.02_	0.66_.02_	N/A	N/A	N/A	N/A	0.64_.04_	0.69_.03_
	Classifier	N/A	.75_.02_	0.75_.02_	0.74_.03_	N/A	N/A	N/A	N/A	0.72_.06_	0.76_.02_

^a^MAE: mean absolute error.

^b^N/A: not applicable.

^c^LR: logistic regression.

^d^-n: with numerical features.

^e^nc: with numerical and categorical features.

^f^nct: with numerical, categorical, and text features.

^g^In the 2-tier task, *F*_1_-pos represents the positive class (high+moderate), and *F*_1_-neg the negative class (low+very low).

## Discussion

### Principal Findings

Our data set was constructed from CDSR by repurposing the quality-of-evidence annotations of human reviewers. The focus of our study is generalized quality assessment in systematic reviews, for which we have proposed a ML method to rate the quality and its associated components for the entire BoE. We have demonstrated that assessment of quality of evidence can be automated with reasonable accuracy when distinguishing between coarse-grained grades of overall quality and when identifying specific quality criteria, including RoB and imprecision. The prediction of less frequent criteria proves more challenging, despite the substantial gains in performance observed when adding more training data. The fine-grained, 4-tier overall grading also turns out to be more challenging than the 2-tier grading; however, the assignment of a quality level is roughly equally good (∼0.5 *F*_1_) across the 4 levels, and on average, the predicted quality is within one level of that assigned by human reviewers.

### Limitations

As we have discussed in the *Consistency of GRADE ratings* section, the findings from user studies examining grading consistency suggest that quality assessment may be subjective to some extent. This is despite the existence of extensive guidelines for GRADE scoring and well-established quality assessment workflows and infrastructure (eg, Cochrane RoB assessment tool). Although the learning outcomes observed here are promising, it is likely that the varying consistency of quality annotations in the data negatively impacts the predictive performance. It would be important to quantify this effect in the future by carrying out a user study that closely matches our task or to develop learning algorithms that are specifically enhanced to deal with label uncertainty. We carried out a brief descriptive analysis of the consistency of RoB assignment, for which we used the labels available in Cochrane reviews (Multimedia Appendix 2) [13].

Furthermore, we have seen that there is value in incorporating lower-level evidence stemming from primary studies, which can additionally increase the quality of assessing the RoB and certain other criteria. However, a limitation here is that alignment is needed between the SoF for a particular outcome and the pool of primary studies that were included. We found this alignment to be noisy in most instances. The exclusion of these cases left us with a reduced number of instances in which the alignments were reliably extracted. A more structured representation of this relationship in the CDSR would help increase the coverage, potentially improving the modeling process. From the perspective of a real-life use case, it would be reasonable to assume that for a given test case, alignment with the relevant primary studies will be available.

Through our ablation analysis for the overall grading of evidence, as well as by removing the entire set of textual features for reason prediction, we have shown that different parts of the systematic review narrative contain quality-related information. When encoded by the NLP component of EvidenceGRADEr, these can be exploited to build a more confident quality assessor. The narrative thus contains complementary signals to nontextual parts of a review. This may be surprising given that the narrative typically provides only a synthesized interpretation of the quality of evidence, and the link to the exact clinical question (PICO criteria) is lost or difficult to extract.

Presently, the system cannot explain its decisions, despite this information playing an important role for a user to decide whether to trust its prediction. A variety of explanation methods could be adopted, for example, gradient-based saliency approaches that reveal which input features contribute the most toward the predicted class [47-49] and are also commonly used in NLP models [50,51]. Another appealing option is counterfactual explanations, which consist of input instances that are closely related to the original instance yet result in a different output class (see the survey by Madsen et al [51] for an overview). These can give the user a sense of direction in which the inputs would need to be changed for the desired prediction to occur (eg, to obtain a higher quality of evidence). They are also generally model-agnostic and can be applied in a post hoc manner. Somewhat orthogonal to these options is joint (or multitask) learning, in which in addition to the original task, the model is trained on another related task, thereby making the decisions on the first task more interpretable. One example of such work is RobotReviewer [14], in which in addition to the article-level RoB labels, the system also predicts for each individual sentence within a full text regardless of whether it was used in assessing the RoB for a particular bias type. Although directly translating this approach to our scenario would be nontrivial (ie, for GRADE criteria, direct quotes from the systematic review are generally not available), there are other possibilities. For example, overall GRADE scoring and prediction of individual downgrading criteria can be performed jointly using a single model. In this way, instead of only providing an overall quality grade, the model would also support the prediction with the downgrading criteria that led to that particular score. We leave the implementation of these options for future work.

### Implications for the Preparation of Systematic Reviews

We envisage that Evidence GRADEr could play a role in different steps in the process of preparing systematic reviews. One possibility is that specific review steps have already been completed, but the review itself has not yet been written. This corresponds to our results obtained with textual features removed (Table 4, under “classifier, -txt”). Concretely, steps in the systematic review process such as the initial screening of primary studies and meta-analysis provide important supporting data for the quality assessment model (eg, the total number of participants for a BoE and a numerical estimate of the relative effect) before the synthesized narrative becomes available. These can be used by the model to obtain the quality grades, which can then be incorporated into the SoF, as well as in the narrative produced by the reviewers. Although the application of our tool further upstream in the reviewing process is possible in principle (eg, before meta-analysis), many strongly predictive inputs (such as the lower and upper CIs) would be missing at test time, thereby negatively impacting predictive performance.

Automated quality grading can also add value to the reviewing process in the form of consistency checking. The reviewers would first perform their GRADE assessment and then use the tool to confirm it. This could be coupled with feedback on features that led to a different predicted score in the case of a discrepancy, which opens up interesting applications regarding explainability, as we discuss at the end of the next section.

### Future Work

There are several possible directions for extending our work in the future. We would like to study the effect of replacing individual review elements (carried out by human experts in our study) with existing automated tools, such as tools for extracting PICO elements or recognizing medical concepts, detecting numerical values, and assigning the RoB to individual studies [12,52-54]. This would represent further progress toward end-to-end systematic review automation, based on systems that make use of pipelines of NLP tools, each modeling individual quality aspects or architectures, thereby addressing multiple constituent tasks simultaneously.

Another possibility is studying the contribution of individual studies and already synthesized data elements toward the overall BoE quality. For example, through counterfactual reasoning [55], we may be able to answer which changes—either at the aggregate level or at the level of primary studies—would lead to higher-quality evidence or to the absence of specific downgrading reasons. This could be of value in guiding the updates of the existing BoE and may highlight how specific aspects of primary research should be carried out.

In terms of end applications of our EvidenceGRADEr, such as in guideline development, knowing not only the overall quality score but also the individual quality criteria would add to transparency and enable the users to better scrutinize the decisions of the system. In the future, we would like to provide explanations for the prediction of quality criteria in addition to the predictions themselves. These explanations could include a selection of, or an abstraction over, the textual snippets from the primary studies, as commonly done in RoB assessment [13]; for other quality criteria, justifications similar to those provided in the footnotes of the SoFs could be generated by the model alongside the predicted quality criteria.

## Appendix

Multimedia Appendix 1 Studies exploring the reliability of rating with Grading of Recommendation, Assessment, Development, and Evaluation.

Multimedia Appendix 2 Analysis: consistency of quality ratings.

Multimedia Appendix 3 Agreement between multiple assigned risk-of-bias judgments in Cochrane reviews as measured using Krippendorff α coefficient.

Multimedia Appendix 4 Descriptive statistics of the textual fields based on all bodies of evidence from the Cochrane Database of Systematic Reviews Quality of Evidence. “μ” is the mean length in tokens; “min” and "max" represent the minimum and maximum length; “% truncated” indicates the number of sections of the given type (in %) that are truncated owing to exceeding the maximum encoding length in BERT (512 tokens).

Multimedia Appendix 5 Experimental details.

Multimedia Appendix 6 Test set results for classification of reasons for downgrading the quality of evidence (T1), reporting precision (P), recall (R), and macroaveraged F1, together with median absolute deviation in subscript across all trials of 10-fold cross-validation. Each reason type was independently trained and evaluated on Cochrane Database of Systematic Reviews Quality of Evidence-supp in a binary setting (eg, risk of bias vs other).

## Abbreviations

BoE: body of evidence
CDSR: Cochrane Database of Systematic Reviews
CDSR-QoE: Cochrane Database of Systematic Reviews Quality of Evidence
GRADE: Grading of Recommendation, Assessment, Development, and Evaluation
LR: logistic regression
ML: machine learning
NLP: natural language processing
PICO: population, intervention, comparison, and outcome
RoB: risk of bias
SoF: summary of findings

## Data Availability

Any requests from third parties to access the data set should be referred first to the Cochrane Collaboration by emailing support@cochrane.org. When Cochrane permits (at its discretion) the use of the data by the third party, Cochrane will grant a license to use the Cochrane Database of Systematic Reviews, including a clause that confirms that Cochrane allows us to grant third party access to the data set created in this work.
