# Supplementary material for: Automating Quality Assessment of Medical Evidence in Systematic Reviews: Model Development and Validation Study
Source: J Med Internet Res. 2023 Mar 13;25:e35568. doi: 10.2196/35568 (PMC10131699; doi:10.2196/35568)
Supplement: Multimedia Appendix 1 [file jmir_v25i1e35568_app1.docx]

| Study | Contains Cochrane reviews | Sample size | Agreement | Agreement on quality criteria | Note |
| --- | --- | --- | --- | --- | --- |
| [24] | yes | 29 reviews | Substantial for 70% of checklist items | - Imprecision: almost perfect  - Indirectness: poor to almost perfect  - RoB: moderate to almost perfect  - Inconsistency: moderate to almost perfect  - Publication bias: fair to almost perfect | Rating according to a proposed checklist of 30 items. |
| [25] | no | 2 reviews | Fair | - Imprecision: fair  - Indirectness: substantial  - RoB: substantial  - Inconsistency: moderate | The rating scheme is not GRADE, but AHRQ EPC. |
| [26] | no | 154 RCTs | Fair to substantial | RoB: fair to substantial | Only evaluate RoB, at the study level only. |
| [21] | exclusively | 4 reviews,  16 outcomes | Substantial | --- | Selected SRs of high methodological quality. Some authors are developers behind GRADE. |
| [20] | yes | 12 reviews,  12 questions | Fair |  | Pilot study to guide the GRADE development using early-stage GRADE guidelines. |
| [27] | exclusively | 37 reviews,  50 questions | Moderate | --- | Use the AHRQ variant of GRADE. Focus on grade stability with the addition of new studies. |
| [28] | exclusively | 100 reviews | Fair | --- | Novice raters without prior experience. |
