# Supplementary material for: Automating Quality Assessment of Medical Evidence in Systematic Reviews: Model Development and Validation Study
Source: J Med Internet Res. 2023 Mar 13;25:e35568. doi: 10.2196/35568 (PMC10131699; doi:10.2196/35568)
Supplement: Multimedia Appendix 2 [file jmir_v25i1e35568_app2.docx]

Multimedia Appendix 2

For risk of bias, we describe our own analysis across studies that appear in multiple Cochrane reviews. We carried out this analysis in a similar manner to [13], i.e. by exploiting the fact that some trials (ranging between 34–98 for each bias component) appear in more than one Cochrane review. We can therefore check for consistency in the RoB assessment of each such trial across the independent assessments of its RoB. We identify studies that occur across multiple systematic reviews based on Google Scholar. Although Google Scholar links are often missing, we refrained from declaring equivalence solely on criteria such as the title out of concern for introducing noise. The results in Multimedia Appendix 3 indicate that there is very high agreement for all bias components. This should probably be seen as an upper bound, as the RoB information from certain studies may simply have been copied from one review to another, and not independently validated. Furthermore, this analysis only sheds light on the reliability of RoB annotations for individual studies, and not for the cumulative BoEs.
