# Supplementary material for: Automating Quality Assessment of Medical Evidence in Systematic Reviews: Model Development and Validation Study
Source: J Med Internet Res. 2023 Mar 13;25:e35568. doi: 10.2196/35568 (PMC10131699; doi:10.2196/35568)
Supplement: Multimedia Appendix 3 [file jmir_v25i1e35568_app3.docx]

Multimedia Appendix 3

| Component | Sample size | α |
| --- | --- | --- |
| allocation concealment | 98 | 0.87 |
| blinding | 36 | 1.00 |
| incomplete output data | 94 | 0.81 |
| other bias | 67 | 0.82 |
| outcome blinding | 34 | 0.94 |
| participant blinding | 34 | 0.95 |
| random sequence generation | 81 | 0.90 |
| selective reporting | 74 | 0.81 |
