# Supplementary material for: Automating Quality Assessment of Medical Evidence in Systematic Reviews: Model Development and Validation Study
Source: J Med Internet Res. 2023 Mar 13;25:e35568. doi: 10.2196/35568 (PMC10131699; doi:10.2196/35568)
Supplement: Multimedia Appendix 4 [file jmir_v25i1e35568_app4.docx]

Multimedia Appendix 4

|  | μ | min | max | % truncated |
| --- | --- | --- | --- | --- |
| full abstract | 799 | 300 | 1835 | 92 |
| abstract conclusion | 103 | 8 | 406 | 0 |
| plain language summary | 456 | 52 | 1219 | 35 |
| authors' conclusions | 464 | 48 | 3828 | 29 |
