# Supplementary material for: Automating Quality Assessment of Medical Evidence in Systematic Reviews: Model Development and Validation Study
Source: J Med Internet Res. 2023 Mar 13;25:e35568. doi: 10.2196/35568 (PMC10131699; doi:10.2196/35568)
Supplement: Multimedia Appendix 5 [file jmir_v25i1e35568_app5.docx]

Multimedia Appendix 5

• BERT pretrained model: scibert_scivocab_uncased;

• BERT tokeniser, with truncate length set to 512;

• Number of layers of FFNN: 3;

• Hidden dimensions in FFNN: 64;

• Activation function in FFNN: relu;

• Embedding dimensionality for categorical features: 32.

The models were trained using the Adam optimiser [1], a batch size of 16, on a single NVIDIA GTX Titan X. All models using the textual inputs were trained for 3 epochs, with the patience criterion set to 1, and the learning rate set to 2e−5. The models using the numerical and categorical inputs only were trained for 20 epochs, with a patience of 5, and the learning rate set to 1e−3.

References:

1. Kingma, D. P. and Ba, J. (2014). Adam: A method for stochastic optimization, arXiv preprint arXiv:1412.6980.
