# Supplementary material for: Automating Quality Assessment of Medical Evidence in Systematic Reviews: Model Development and Validation Study
Source: J Med Internet Res. 2023 Mar 13;25:e35568. doi: 10.2196/35568 (PMC10131699; doi:10.2196/35568)
Supplement: Multimedia Appendix 6 [file jmir_v25i1e35568_app6.docx]

Multimedia Appendix 6

|  | GRADE downgrading criteria | | | | | | | | | | | | | | |
| --- | --- | --- | --- | --- | --- | --- | --- | --- | --- | --- | --- | --- | --- | --- | --- |
|  | RoB | | | Imprecision | | | Inconsistency | | | Indirectness | | | Publication bias | | |
| BERT variant | P | R | F1 | P | R | F1 | P | R | F1 | P | R | F1 | P | R | F1 |
| SciBERT | .68 | .93 | .78_.03_ | .66 | .86 | .75_.01_ | .3 | .37 | .31_.08_ | .41 | .44 | .41_.07_ | .4 | .42 | .39_.12_ |
| BERT-base-uncased | .66 | .94 | .77_.02_ | .64 | .89 | .74_.01_ | .25 | .35 | .29_.06_ | .39 | .49 | .42_.09_ | .29 | .35 | .29_.16_ |
| BioMed-RoBERTa-base | .69 | .92 | .79_.03_ | .66 | .87 | .74_.02_ | .27 | .43 | .33_.02_ | .41 | .53 | .45_.13_ | .33 | .43 | .35_.27_ |
